# Identification and functional analysis of novel phosphorylation sites in the RNA surveillance protein Upf1

**DOI:** 10.1093/nar/gkt1049

**Published:** 2013-11-05

**Authors:** Clarivel Lasalde, Andrea V. Rivera, Alfredo J. León, José A. González-Feliciano, Luis A. Estrella, Eva N. Rodríguez-Cruz, María E. Correa, Iván J. Cajigas, Dina P. Bracho, Irving E. Vega, Miles F. Wilkinson, Carlos I. González

**Affiliations:** ^1^Department of Biology, University of Puerto Rico-Río Piedras Campus, San Juan, PR, ^2^Department of Reproductive Medicine, University of California, San Diego, CA, ^3^Department of Biochemistry, University of Puerto Rico-Medical Sciences Campus, San Juan, PR and ^4^Molecular Sciences Research Building, San Juan, PR

## Abstract

One third of inherited genetic diseases are caused by mRNAs harboring premature termination codons as a result of nonsense mutations. These aberrant mRNAs are degraded by the Nonsense-Mediated mRNA Decay (NMD) pathway. A central component of the NMD pathway is Upf1, an RNA-dependent ATPase and helicase. Upf1 is a known phosphorylated protein, but only portions of this large protein have been examined for phosphorylation sites and the functional relevance of its phosphorylation has not been elucidated in *Saccharomyces cerevisiae*. Using tandem mass spectrometry analyses, we report the identification of 11 putative phosphorylated sites in *S. cerevisiae* Upf1. Five of these phosphorylated residues are located within the ATPase and helicase domains and are conserved in higher eukaryotes, suggesting a biological significance for their phosphorylation. Indeed, functional analysis demonstrated that a small carboxy-terminal motif harboring at least three phosphorylated amino acids is important for three Upf1 functions: ATPase activity, NMD activity and the ability to promote translation termination efficiency. We provide evidence that two tyrosines within this phospho-motif (Y-738 and Y-742) act redundantly to promote ATP hydrolysis, NMD efficiency and translation termination fidelity.

## INTRODUCTION

Eukaryotic gene expression is highly regulated to guarantee fidelity in the conversion of genetic information into biological function. Several mechanisms are responsible for maintaining fidelity during the flow of genetic information. One such mechanism is the nonsense-mediated mRNA decay (NMD) pathway, which recognizes and degrades mRNAs that contain premature translation termination codons (PTCs), thereby preventing the synthesis of truncated proteins (1–6). This surveillance pathway also contributes to cellular homeostasis by regulating the expression of ∼3–20% of the transcriptome of eukaryotes across the phylogenetic scale (7–12).

The core factors essential for NMD in all organisms reported on to date are the UP-frameshift 1 (Upf1), Upf2 and Upf3 proteins. Upf1 is a predominantly cytoplasmic RNA-binding protein that exhibits RNA-dependent ATPase and RNA helicase activities that are essential for NMD (13–20). The RNA-dependent ATPase activity of Upf1 is triggered by the formation of a ‘surveillance complex’ comprised of all three Upf proteins (6,20–24). Several models have been proposed to explain how these components of the NMD machinery recognize a PTC and recruit RNA degradation proteins (25–39). Most models revolve around the notion that RNA decay is triggered when a stop codon is followed by a second signal that defines the stop codon as premature (3,11). In the ‘*faux* 3′-UTR’ model, translation termination at a normal stop codon is proposed to be fundamentally different from translation termination at a PTC; mRNA decay is activated by the aberrant nature of premature termination (27). According to this model, proper termination requires an interaction between a terminating ribosome and a specific messenger ribonucleoprotein (mRNP) structure localized 3′ to the stop codon (27,40,41). The ‘*faux* 3′-UTR’ model further proposes that the proximity of the poly(A) binding (Pab1) protein to the PTC is important for NMD activation (27). In mammals, a well-established NMD second signal is an exon–exon junction downstream of a stop codon, as this allows a protein complex recruited at exon–exon junctions—the exon junction complex (EJC)—to activate NMD (31,33,42). When a translating ribosome encounters a PTC upstream of an EJC, a SURF complex (SMG1C:Upf1:eRF1:eRF3) is assembled (36,39,43–45), which, in turn, recruits both mRNA decapping and degradation enzymes (11,46).

Upf1 is a phosphoprotein, which has led investigators to examine whether Upf1 phosphorylation has roles in NMD (1,39,47,48). Previous studies have provided evidence that phosphorylation and dephosphorylation cycles of Upf1 promote NMD in *Caenorhabditis elegans* and mammals (39,47,49–51). While the underlying mechanism remains to be fully established, it is known that Upf1 is phosphorylated by the NMD factor, SMG-1, when the SURF:ribosome complex interacts with the EJC (36,44), ultimately leading to the degradation of PTC-containing mRNAs (11,46). To understand the molecular role of Upf1 phosphorylation in NMD, it is critical to identify the phosphorylated amino acids in Upf1. To date, few phospho-amino acids have been identified in Upf1. In mammalian UPF1, two phosphorylated serine residues at the C-terminus (S-1078 and S-1096) and one at the N-terminus (T-28) have been identified (39,48). *Saccharomyces cerevisiae* Upf1 has been shown to be a phosphoprotein, but the identity of its phosphorylated residues is not known (1).

In this study, we have used mass spectrometry analysis to identify 11 novel phosphorylation sites in *S. cerevisiae* Upf1. Five of the phosphorylated residues are conserved in *Arabidopsis, Drosophila melanogaster*, *C. elegans* and human UPF1. Our structure–function analysis revealed the existence of a ‘phospho-motif’ harboring phosphorylated residues in the C-terminus that is essential for the ability of Upf1 to function in NMD, as well as its ATP hydrolysis function and its ability to promote translation termination efficiency. Mutation of two of the tyrosines in this phospho-motif decreased Upf1’s NMD and ATPase hydrolysis activities, as well as Upf1’s ability to promote translation termination accuracy. Our studies provide a foundation for future studies to determine the precise molecular roles of phospho-amino acids in the Upf1 NMD protein.

## MATERIALS AND METHODS

### Yeast strains and plasmids

*Saccharomyces cerevisiae* strain W303 *upf1Δ* (*MATa ade2-1 his3-11,15 leu2-3,112 trp1-1 ura3-1 can1-100 upf1::*HIS3 NMD2 UPF3) was used as the wild-type (WT) strain for all the experiments described in this study. The yeast 2μ plasmid pG-1 containing the FLAG-*UPF1* allele was used as the vector (52). The *upf1* deletions used in these experiments were constructed by single-step cloning using the FLAG-*UPF1* allele as the template (53). The *upf1* point-mutants were constructed by site-directed mutagenesis using the FLAG-*UPF1* allele as the template. Yeast transformations were performed by the lithium acetate method (54).

### Whole cell protein extracts and Western blotting

Cells were grown in 30-ml culture to mid-log phase (OD_600_ = 0.7–0.8) and lysed using glass beads in lysis buffer (50 mM Tris-HCl pH 7.5, 100 mM NaCl, 5 mM EDTA, 0.01% Triton-100X, 10% Glycerol) containing 1 mM phenylmethylsulfonyl fluoride and 1X protease inhibitor cocktail (Sigma). Total protein extracts were quantified using the BioRad protein assay with bovine serum albumin (BSA) as a protein standard. Protein (20 µg) was loaded into each lane and resolved by 10% SDS-polyacrylamide gel electrophoresis and transferred to a nitrocellulose membrane. Flag-Upf1 was detected by Western blot using anti-Flag as primary antibody (Sigma) and anti-Mouse peroxidase conjugated (Sigma) as secondary antibody. Pab1 was used as a loading control. Western blot signals were exposed using SuperSignal West Dura chemiluminescent substrate (Thermo Scientific).

### Upf1 protein purification

WT and mutant forms of Upf1 were purified as FLAG fusion proteins from yeast using anti-FLAG M2 affinity gel (Sigma), as previously described (1). Briefly, cells were grown in 500-ml culture to an optical density (OD_600_) of 0.8–0.9 and lysed with glass beads in buffer XA (50 mM Tris-HCl pH 7.5, 150 mM NaCl, 5 mM EDTA, 0.01% Triton-100X, 10% Glycerol) containing 1 mM phenylmethylsulfonyl fluoride and 1X protease inhibitor cocktail (Sigma). For tandem mass spectometry analysis, the following phosphatase inhibitors were added to buffer XA: 30 mM Sodium Fluoride, 30 mM β-glycerol phosphate, 5 mM Sodium Pyrophosphate and 100 nM Okadaic acid. After the anti-FLAG affinity gel was equilibrated with TBS buffer, the extract was added to the affinity gel and both were incubated overnight at 4°C with constant rocking. The beads were washed with 50 ml of buffer XB (buffer XA containing 250 mM NaCl) and then washed with 50 ml of buffer XA. Bound protein was eluted using buffer XC (buffer XA containing 5 μg/μl of Flag Peptide; Sigma). Eluted proteins were resolved by 10% SDS-PAGE, stained with Coomassie blue, and analysed by Western blot. Protein concentration was determined from Coomassie blue-stained gels using BSA as a protein standard.

### Tryptic digestion and tandem mass spectrometry (MS/MS)

Tryptic digestion of purified Flag-Upf1 protein was performed as described by Vega *et al.* (55). Briefly, the 10% SDS-PAGE was equilibrated in deionized water and Flag-Upf1 gel fragment was excised and unstained using 100 mM ammonium bicarbonate (Ambic); 50% acetonitrile (ACN). The solvent was removed and the gel slice was incubated with 100% ACN at room temperature, followed by vacuum drying. Then, the gel slide was resuspended in digestion buffer (DB) (50 mM Ambic; 10% ACN) mixed with 1 µg of trypsin (Promega), and incubated overnight at 37°C. To elute the sample, the gel slice was incubated in 50% ACN; 5% formic acid for 1 h. The obtained peptides were dried by speed vacuum and resuspended in Loading Solvent (0.1% formic acid, 1% ACN in HPLC-graded water).

For mass spectrometry analysis, the Proteome X LTQ (Thermo Electron) mass spectrometer was used. The peptides were loaded to and eluted from a ZipTip-C18 column (Thermo Fisher Scientific) on line with the mass spectrometer. Parameters used for mass spectra analysis are described in Vega *et al.* (55). Acquired MS/MS (MS2) spectra were analysed using the Thermo Electron Bioworks Browser and the SEQUEST algorithm. Data were analysed using non-redundant protein database considering the mass increase of (+79.99) based on addition of a phosphate group.

### RNA isolation and Northern blot analysis

Total RNA was isolated using the hot phenol method (56) and mRNA abundance was determined by Northern blotting (1,26). Random-primed DNA probes were prepared from a 0.6-kb EcoRI-HindIII fragment spanning a region of the *CYH2* mRNA. Northern blots were quantitated using a BioRad Molecular Imager FX. The activity of NMD was calculated by comparing the ratio of *pre-CYH2* to *CYH2* in *upf1*Δ strains transformed with either a vector (0%) or WT Upf1 (100%). The values shown represent the average value ± standard deviation from three independent experiments.

### ATPase activity assay

ATP hydrolysis was monitored using a charcoal assay as previously described (19,57). Reactions were carried out in a total volume of 20 μl which contained 50 mM Tris-HCl (pH 8.0), 50 mM KCl, 3 mM MgCl_2_, 1 mM DTT, 100 μM poly(T), 100 μM ATP, 1 uCi of [(^32^P] ATP (3000 Ci/mmol) and 5 ng of Upf1 protein. After incubation for 20 min at room temperature, reactions were stopped and unreacted ATP was absorbed by addition of 200 μl of 5% charcoal in 20 mM phosphoric acid. The charcoal was pelleted by centrifugation for 10 min at 13 200 *g*, and the amount of ^32^PO_4_ released was determined by counting the radioactivity in a 100-μl aliquot of the supernatant in a scintillation counter. To determine background, five controls were performed without Upf1 protein for each experiment. Values shown represent averages from three independent experiments. Statistical analysis was performed using GraphPad Prism 5 software.

### Dual luciferase assays

Dual luciferase assays were performed with the dual luciferase reporter assay system (Promega) as described previously with minor modifications (58,59). Cells were grown to mid-log phase (OD_600_ = 0.8) in SC-Ura medium. Ten microliters of cells were removed from the culture and transferred to 100 μl of 1× passive buffer. Cells were lysed for 15 s and 10 μl were used for luminescence measurements using a TD-20/20 luminometer (Tuner Designs). The following steps were used for luminescence measurements: 10 μl of the firefly luciferase reagent (LARII) were added to the sample with a 2 s equilibration time, and measurement of luminescence with a 10 s integration time, followed by addition of 10 μl of the *Renilla* luciferase reagent and firefly quenching (Stop & Glow), 2 s equilibration time and measurement of luminescence with a 10 s integration time. The values shown represent the ratio of the firefly luciferase activity to the *Renilla* activity of the stop codon-containing constructs divided by the ratio of the firefly luciferase activity to the *Renilla* activity of the sense codon-containing construct multiplied by 100. For each strain, at least three independent transformants were assayed and each individual extract was scored for luminescence activity in triplicates. Statistical analysis was performed using GraphPad Prism 5 software.

## RESULTS

### Identification of 11 novel phosphorylation sites in yeast Upf1

**Figure 1.**
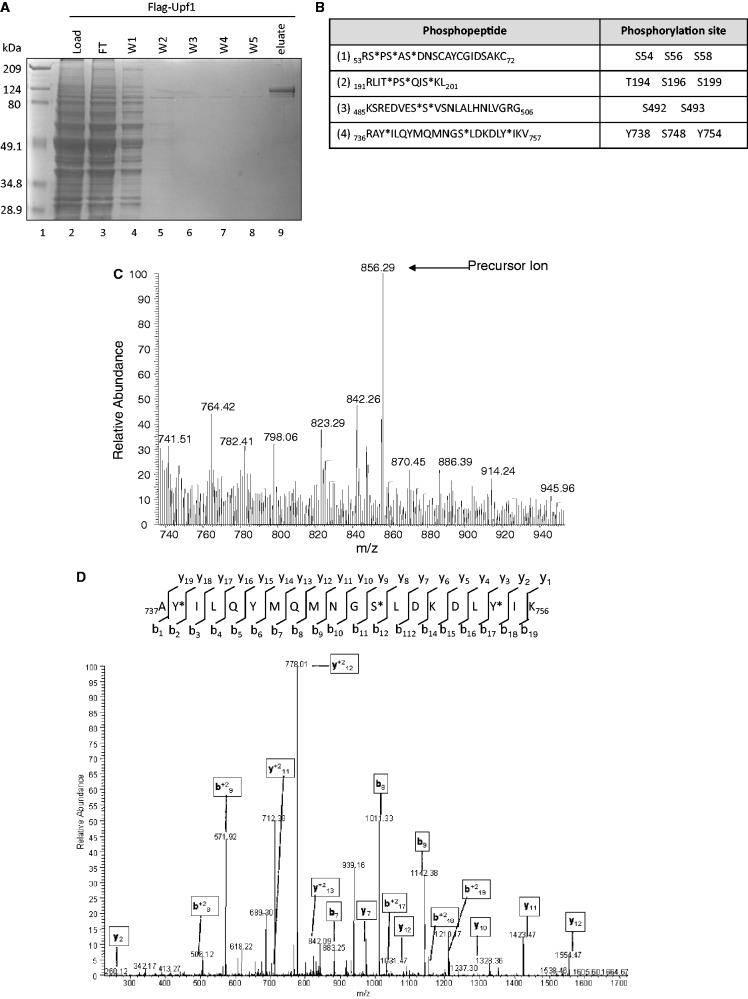
Identification of novel phosphorylation sites in Upf1. (**A**) Coomassie blue-stained 10% SDS-PAGE of immunopurified Flag-Upf1 protein. FT = flowthrough, W1–W5 = washes 1–5. (**B**) Immunopurified Flag-Upf1 protein was in-gel digested with trypsin. The peptides produced were resolved by LTQ linear ion trap mass spectrometry (Proteome X LTQ Workstation,Thermo) and analysed as described in Materials and Methods section. Asterisk (*) shows the Upf1 phosphorylated residues. (**C**) The arrow on the MS spectrum points toward the charged precursor ion corresponding to phosphopeptide 4. (**D**) This precursor ion was selected and subjected to fragmentation (MS2), generating *b* and *y* product ions that represent specific fragments used for identification of the peptide sequence and phosphorylation sites. The asterisks (*) illustrate phosphorylated amino acids.

Our previous studies demonstrated that *S. cerevisiae* Upf1 is a phosphoprotein (1). To identify the phosphorylated residues, we purified *S. cerevisiae* Upf1 protein from an *upf1Δ* strain harboring a plasmid encoding a Flag-tagged *UPF1* allele. Purification of Flag-tagged Upf1 protein was carried out in the presence of phosphatase inhibitors to maintain the phosphorylation of its amino acid residues. The purified sample was resolved by 10% SDS-PAGE and stained with Coomassie blue (Figure 1A, lane 9). Flag-Upf1 migrated in the 80–124 kDa region, consistent with previous reports (13,18,60).

**Figure 2.**
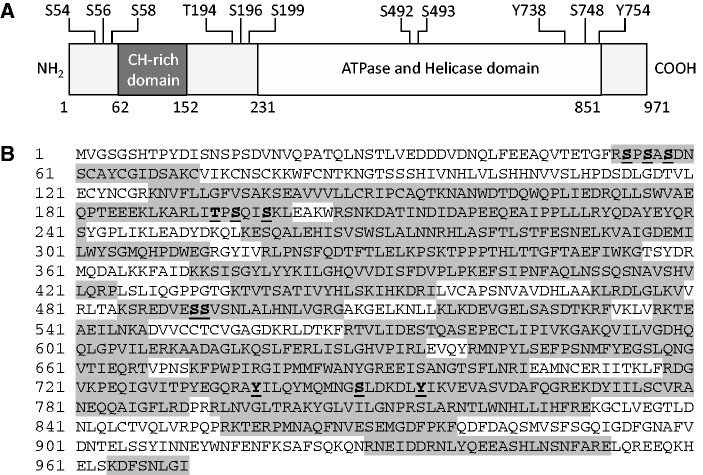
Eleven phosphorylation sites were identified in Upf1. (**A**) Schematic representation of the Upf1 protein depicting the locations of the cysteine- and histidine-rich (CH-rich) domain, ATPase domain and helicase domain. The localization of the 11 phosphorylation sites identified by MS/MS in Upf1 protein is represented. (**B**) Amino acid sequence of Upf1 protein in *S. cerevisiae*. Sixty-seven percent (67%) sequence coverage was achieved by tandem MS/MS. Sequence coverage is depicted in gray, and phosphorylated residues are underlined.

We excised the Coomassie-stained band that corresponded to Upf1 protein (∼109 kDa) and subjected it to trypsin digestion. The resulting tryptic peptides were eluted and analysed by mass spectrometry (see Materials and Methods section). The identity of the peptides and the sites of phosphorylation were determined by correlating MS2 spectra with sequences from the NCBI non-redundant protein database using the SEQUEST algorithms, and searching parameters that included differential mass modification due to phosphorylation (61,62). From this analysis, four phosphopeptides were identified in yeast Upf1: phosphopeptide 1 (_53_RSPSASDNSCAYCGIDSAKC_72_), phosphopeptide 2 (_191_RLITPSQISKL_201_), phosphopeptide 3 (_485_KSREDVESSVSNLALHNLVGRG_506_) and phosphopeptide 4 (_736_RAYILQYMQMNGSLDKDLYIKV_757_) (Figure 1B). The corresponding full MS and MS2 spectra for these phosphopeptides were also manually verified by detecting the neutral loss of HPO_4_ (80 Da) or H_3_PO_4_ (98 Da) after ionization. Phosphopeptide 1 corresponds to amino acids 53–72 and contains three phosphorylated residues: S-54, S-56 and S-58 (Figure 1B). Phosphopeptide 2 corresponds to amino acids 191–201, of which T-194, S-196 and S-199 were found to be phosphorylated (Figure 1B). Phosphopeptide 3 corresponds to amino acids 485–506 and harbors two phosphorylated residues: S-492 and S-493 (Figure 1B). Finally, phosphopeptide 4 corresponds to amino acid residues 736–757, of which Y-738, S-748 and Y-754 were found to be phosphorylated (Figure 1B). In sum, 11 phosphorylated residues were identified in *S. cerevisiae* Upf1 (Figure 2A). Figure 1C and D show an example of the MS and MS2 spectra corresponding to phosphopeptide 4. The MS2 spectrum shows the identification of *b* and *y* ions produced from the fragmentation of its precursor ion (*m/z* = 856.29). The non-phosphorylated forms of phosphopeptides 1–4 were also observed in this analysis. A total of 291 peptides distributed throughout Upf1 protein were identified by our MS/MS analysis (Figure 2A). These peptides included 652 of the 971 amino acids in yeast Upf1 (67% sequence coverage) (Figure 2B).

**Figure 3.**
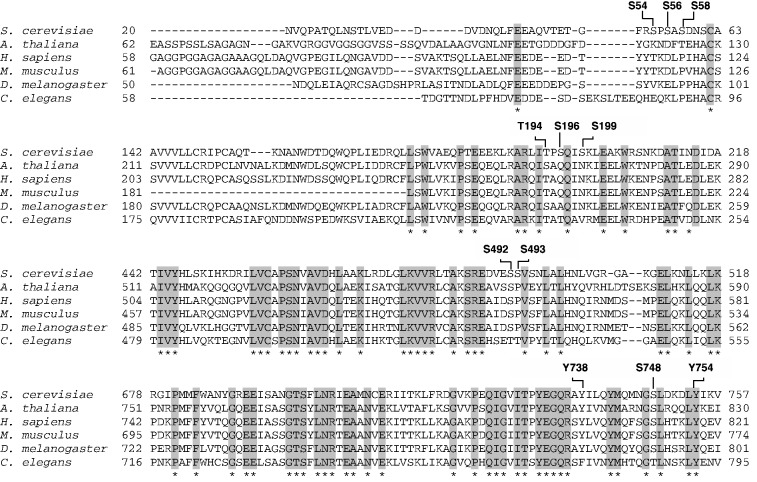
Phosphorylated residue within the ATPase and helicase domain of Upf1 is completely conserved. Sequence alignment of *S. cerevisiae* (NCBI NP_013797), *H. sapiens* (NCBI AAC26788), *M. musculus* (NCBI EDL28813), *A. thaliana* (NCBI AAL92018), *D. melanogaster* (NCBI AAF48115.2) and *C. elegans* (NCBI AAC26789) shows complete conservation of one phosphorylated residue, Y754F. This alignment was constructed with CLUSTALW using T-Coffee program (version 9.02) (63). Completely conserved sequences are shown in gray.

Sequence alignment of lower and higher eukaryotes shows complete conservation of the phosphorylated Upf1 residue Y-754, suggesting an important biological role for this amino acid (Figure 3). The amino acids corresponding to *S. cerevisiae* Upf1 T-194, S-492, Y-738 and S-748 were similar in the *Homo sapiens*, *Mus musculus*, *D. melanogaster*; *Arabidopsis thaliana and C. elegans* orthologs of Upf1. Of note, most of these phosphorylated residues are located within the ATPase and helicase domain (Figures 2A and 3), which has been shown to be critical for NMD activity (13,14,18–20).

### An Upf1 motif harboring three novel phosphorylated residues is required for NMD and translation termination

**Figure 4.**
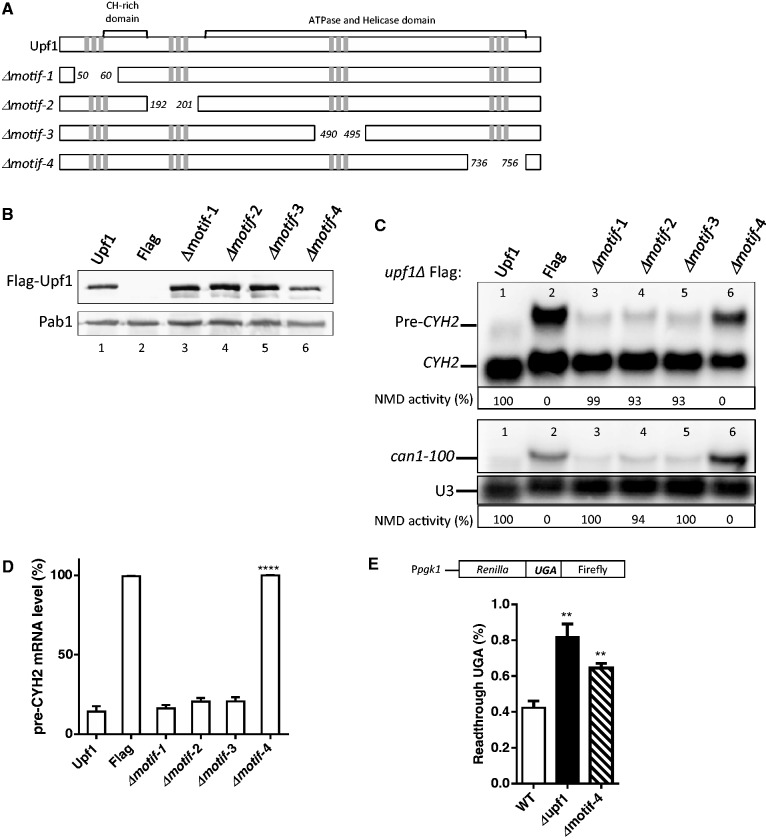
The phosphorylated motif-4 of Upf1 is essential for NMD. (**A**) Schematic representation of Upf1 mutants. Phosphorylated residues are represented as gray rectangles. In motif 1 amino acids T50–N60 were deleted, in motif 2 amino acids L192–L201 were deleted, in motif 3 V490–S495 were eliminated and in motif 4 residues R736–K756 were eliminated. (**B**) Western blot analysis of cytoplasmic extracts demonstrating Upf1 protein expression. Poly(A) binding protein (Pab1) was used as a loading control. (**C**) NMD activity of deletions of Upf1 phosphorylated motifs were determined by Northern blot analysis of total cellular RNA. (**D**) Mean value ± standard deviation of *pre-CYH2* mRNA accumulation from Northern blot in (C). (**E**) Dual luciferase assay was conducted to determine the efficiency of UGA stop codon recognition. Two-tailed *t*-tests were used for statistical analysis. The asterisk indicates a statistical significant (*P* < 0.05) result when compared to the WT strain.

Since the phosphorylated amino acids that we identified in Upf1 are clustered in four small regions (Figure 4A), we elected to first test their importance by independently deleting these four regions. The four ‘phospho-motifs’ are: motif-1 (T50-N60), motif-2 (L192-L201), motif-3 (V490-S495) and motif-4 (R736-K756). Constructs harboring deletions of these Upf1 phospho-motifs were generated and transformed into an *upf1Δ* strain (Figure 4A). WT *upf1* and the Flag-vector alone served as controls. The upf1 mutant proteins were expressed at levels comparable to that of WT Upf1 as shown by Western blotting (Figure 4B). NMD activity was determined by using Northern blot analysis to detect two NMD substrate mRNAs: *CYH2* pre-mRNA and *can1-100* mRNA. The former is a NMD substrate by virtue of having in-frame premature terminations codons in the intron (64) and thus we used *CYH2* pre-mRNA-to-mature *CYH2* mRNA ratio as a measure of NMD activity. We measured *can1-100* mRNA level by normalizing against the *U3* mRNA loading control. Analysis of total cellular RNA from WT and mutant strains revealed that three of the deletion strains complemented the NMD defect in the *upf1Δ* strain as well as WT *UPF1* (Figure 4C and D, compare lane 1 with lanes 3–5). This indicated that these deletions did not measurably affect NMD activity. In contrast, the deletion-mutant lacking phospho-motif-4 (*Δmotif-4*) was not able to complement the NMD defect (Figure 4C and D, lane 6). This Upf1 phospho-motif contains the highly conserved phosphorylated residue, Y-754, as well as two other phosphorylated amino acids: Y-738 and S-748 (Figure 3).

Another role of Upf1 is its ability to promote translation termination fidelity, an activity that is separable from its role in NMD (65). To test the role of phospho-motif-4 in translation termination efficiency we used a well-established dual luciferase assay (66). This assay consists of a bicistronic reporter mRNA that contains the *Renilla* and firefly luciferase open reading frames in tandem, separated by a linker sequence that harbors a stop codon (Figure 4E). In yeast strains defective in translation termination, higher levels of firefly luciferase are synthesized due to a higher frequency of ribosome read-through of the stop codon. Therefore, an increase in the activity of the firefly luciferase relative to the *Renilla* activity is directly proportional to defects in translation termination. Using this dual luciferase assay we quantitated read-through of the UGA stop codon, which has been reported to show higher levels of read-through in the *upf1Δ* strain compared to UAG and UAA stop codons (67). As previously reported, the *upf1Δ* strain expressed higher levels of firefly luciferase compared to the WT strain (Figure 4E) (67,68). The deletion-mutant lacking phospho-motif-4 was not able to rescue this defect, indicating that this motif has a role in translation termination efficiency. Together, these results suggest that the phospho-motif-4 of Upf1 plays a role in both NMD activity and translation termination accuracy.

### Upf1 Y-738 and Y-742 function in NMD and translation termination accuracy

**Figure 5.**
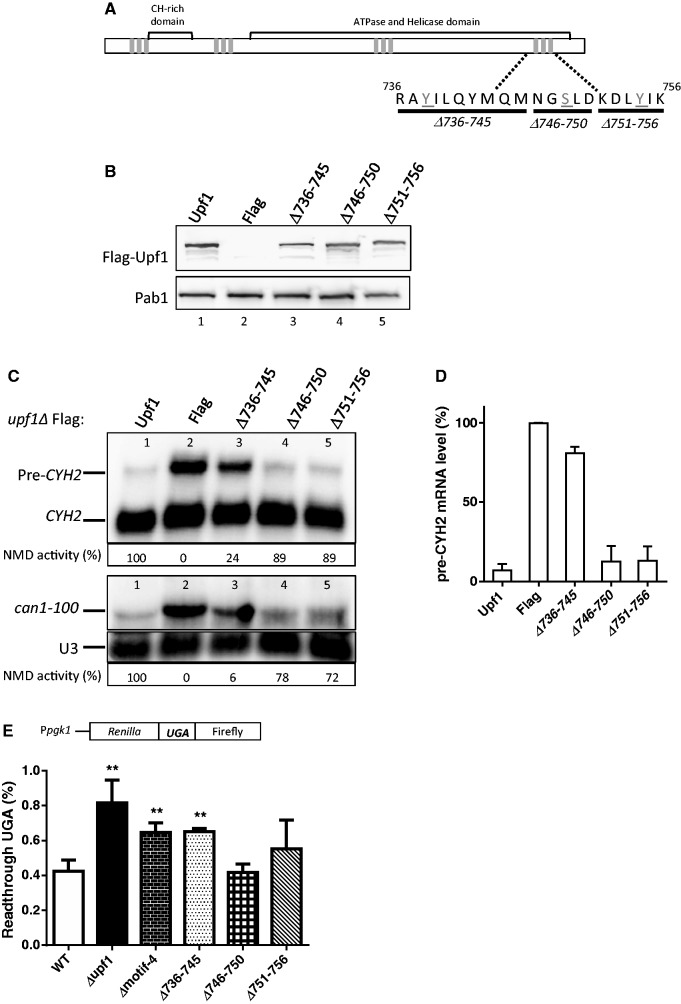
Upf1 region encompassing amino acids 736–745 is essential for NMD. (**A**) Schematic representation of Upf1 indicating the deleted amino acids within the motif-4. Each deletion contains a phosphorylated residue (underlined). (**B**) Western blot analysis of cytoplasmic extracts demonstrating Upf1 protein expression. Poly(A) binding protein (Pab1) was used as a loading control. (**C**) NMD activity of Upf1 phosphorylated motif-4 deletions determined using Northern blot analysis of total cellular RNA. (**D**) Mean value ± standard deviation of *pre-CYH2* mRNA accumulation from Northern blot in (C). (**E**) Dual luciferase assay was conducted to determine the efficiency of UGA stop codon recognition. Two-tailed *t*-tests were used for statistical analysis. The asterisk indicates a statistical significant (*P* < 0.05) result when compared to the WT strain.

To dissect the sequences within phospho-motif-4 required for NMD activity, we used PCR-mediated mutagenesis to generate three additional deletion mutants, each of which harbor a conserved phosphorylated residue identified by our MS analysis (Figure 5A). Expression of these *upf1* mutants was confirmed by Western blotting (Figure 5B). Northern blot analysis was conducted on total cellular RNA isolated from the WT and mutant strains, and NMD activity was determined as described above. This revealed that deletion of residues 736–745 reduced NMD activity as measured by both *pre-CYH2* mRNA (24%) and *can1-100* (6%) mRNA levels (Figure 5C and D, lane 3). In contrast, deletion of amino acids 746–750 or 751–756 did not impair NMD activity (Figure 5C, lanes 4 and 5). Using the dual luciferase assay described above, we also assessed whether these three deletion mutants had a defect in promoting translation termination fidelity. We found that only the Δ736–745 strain expressed significantly higher levels of firefly luciferase as compared to the WT strain, indicating higher percentages of read-through (Figure 5E). Together, these results indicated that the phosphorylated region encompassing amino acids 736–745 is specifically required for both Upf1’s NMD activity and its ability to promote translation termination accuracy.

**Figure 6.**
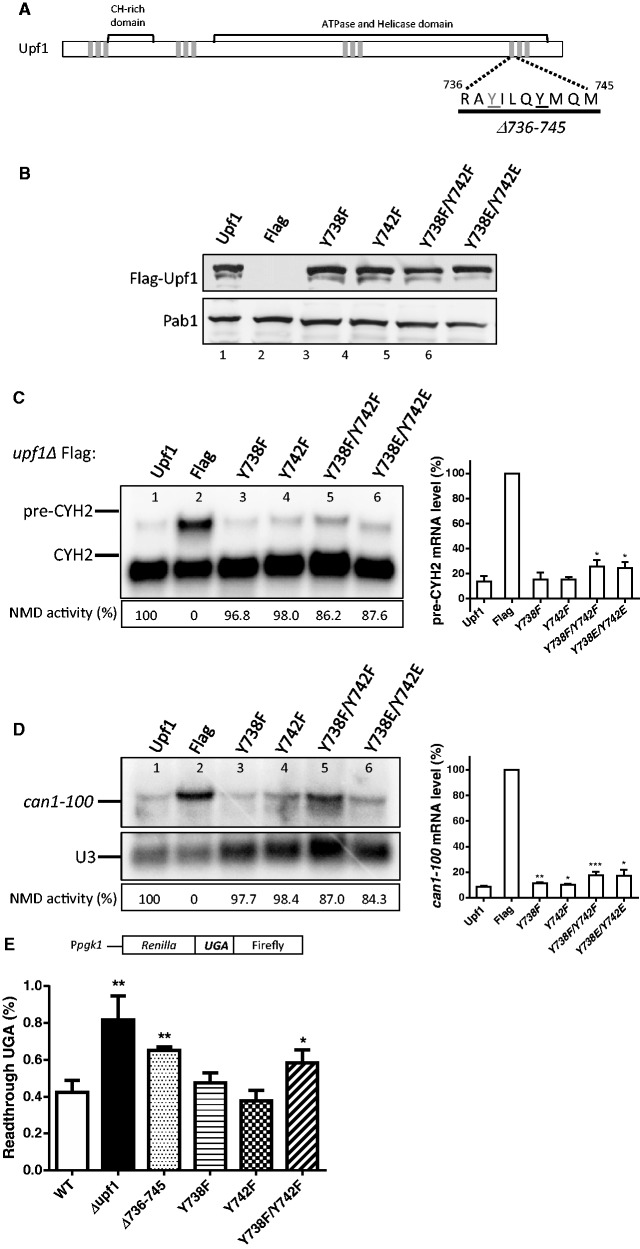
The Upf1 phosphorylated Y-738 residue and the adjacent Y-742 are important for NMD activity. (**A**) Schematic representation of Upf1 indicating the deleted amino acids in the 736–745 region. The Y738 phosphorylated residue is underlined in gray, and the potential phosphorylated residue, Y742, is underlined in black. (**B**) Western blot analysis of cytoplasmic extracts demonstrating Upf1 protein expression. Poly(A) binding protein (Pab1) was used as a loading control. (**C**) (Left Panel) NMD activity of Upf1 phosphorylated residues within 736–745 region measured by *pre-CYH2* to mature mRNA ratio and determined by Northern blot analysis of total cellular RNA. (Right Panel) Mean value ± standard deviation of *pre-CYH2* mRNA accumulation from Northern blot. (**D**) (Left Panel) NMD activity of Upf1 phosphorylated residues within 736–745 region measured by *can1-100* mRNA levels and determined by Northern blot analysis. (Right Panel) Mean value ± standard deviation of *can1-100* mRNA accumulation from Northern blot. (**E**) Dual luciferase assay was conducted to determine the efficiency of UGA stop codon recognition. Two-tailed *t*-tests were used for statistical analysis. The asterisk indicates a statistical significant (*P* < 0.05) result when compared to the WT strain.

Inspection of the amino acids 736–745 region revealed that, in addition to the tyrosine that we found was phosphorylated, Y-738 (Figure 2A), another tyrosine is also present: Y-742 (Figure 5A). While Y-742 was not identified as a phosphorylated amino acid in our MS/MS analysis, it may be phosphorylated under some circumstances or at levels below the detection limits of our analysis (Figure 1B). To test the functional role of Y-738 and Y-742, we used site-directed mutagenesis to mutate them (Figure 6A). These tyrosine residues were mutated to phenylalanine to mimic a non-phosphorylated form of tyrosine and they were mutated to glutamic acid to mimic a constitutively phosphorylated form of tyrosine. All mutant proteins were expressed at virtually identical levels, suggesting they exhibit similar stability (Figure 6B). Northern blot analysis was conducted on total cellular RNA isolated from WT and mutant strains, and NMD activity was determined essentially as described above. We found that the single-point mutants, Y738F and Y742F, fully rescued NMD activity of a chromosomal *UPF1* deletion-mutant strain, indicating that they are not compromised in their ability to function in NMD (Figure 6C and D, lanes 3 and 4). In contrast, the Y738F/Y742F and Y738E/Y742E double-point mutants had a modest but reproducible inability to fully rescue NMD activity, as measured by both *pre-CYH2* to mature mRNA ratio and *can-100* mRNA levels (Figure 6C and D, lanes 5 and 6). Likewise, the Y738F/Y742F double-point mutant exhibited increased translational read-through activity (Figure 6E). Taken together, these data pinpoint a small phosphorylated region of Upf1, consisting of amino acids 736–745, as being critical for its function in both NMD and promotion of translation termination accuracy. These two activities were perturbed when both tyrosine residues within this region were converted into phenylalanine, suggesting that the phosphorylation of both tyrosines serves in a redundant manner.

### Y-738 and Y-742 are critical for Upf1’s ATPase activity

**Figure 7.**
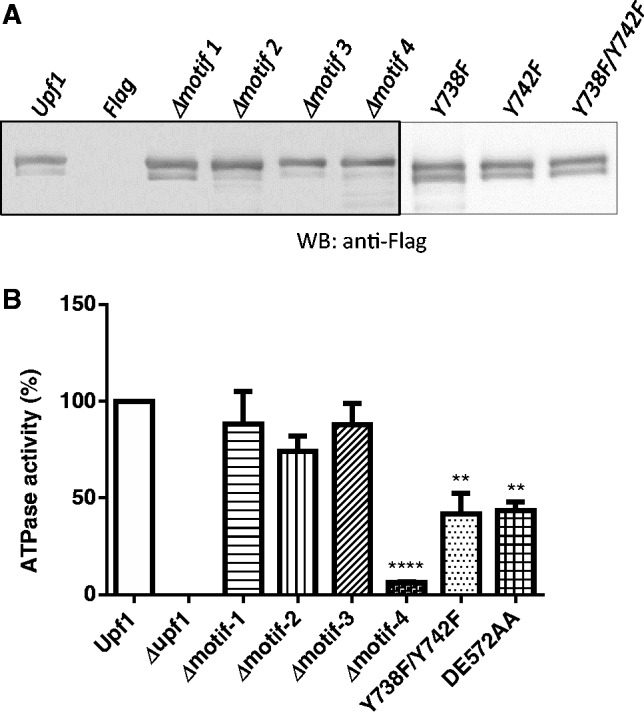
The ATP hydrolysis of Upf1 is inhibited in phosphorylation mutants. WT and mutant form of the Upf1 protein was purified as described in Materials and Methods section. (**A**) Purified proteins were analysed by SDS-PAGE and analysed by Western blotting using monoclonal antibody against Flag epitope (Sigma). (**B**) The ATPase activities of the WT and mutant form of Upf1 were determined using 5 ng of purified proteins (see Materials and Methods section). The Upf1 ATPase mutant DE572AA was used as a control.

Previous studies have demonstrated that Upf1’s ATPase and helicase activities are essential for its NMD function (18,36,69). Recently, it was demonstrated that the ATPase activity of Upf1 stimulates the removal and recycling of NMD factors from PTC-containing mRNPs (21,69). An outstanding issue has been whether Upf1’s ATPase activity is dictated by its phosphorylation status. This is important given that considerable evidence suggests that a cycle of phosphorylation and dephosphorylation is required for Upf1 to function in NMD (11,70). Therefore, we examined whether phosphorylation affects Upf1’s ATPase activity. For these experiments, we immunopurified WT and *upf1* mutant proteins expressed from Flag-tagged plasmids. The recombinant proteins were resolved by 10% SDS-PAGE and analysed by Western blot using anti-Flag antibody (Figure 7A). The ATPase activity of the purified *upf1* mutant proteins was monitored using an ATPase charcoal assay and compared to the ATPase activity of the WT Upf1 protein (13,18). This analysis revealed that deletion of phospho-motif-4 almost completely abolished Upf1’s ATPase activity (to 6% of the control; Figure 7B). In contrast, the previously described ATPase domain mutant—DE572AA (18)—had only modestly reduced ATPase activity compared to WT Upf1 (Figure 7B). The Upf1 mutants lacking phospho-motifs-1, -2 or -3 had normal ATPase activity. To specifically examine the role of the two tyrosines within phospho-motif-4, we tested the Y738F/Y742F double-point mutant (Figure 6A) and found that it exhibited significantly reduced Upf1 ATPase activity (Figure 7B). The Y738F/Y742F mutant had 41% of the activity of WT Upf1, which was a less severe defect than that exhibited by the phospho-motif-4-deletion mutant, but comparable with the well-established DE572AA ATPase mutant. Taken together, our results indicate that phospho-motif-4 has an essential role in Upf1’s ATPase activity that is mediated, in part, by two tyrosine residues within this motif.

## DISCUSSION

Despite the considerable progress in understanding the role of Upf1 phosphorylation in NMD (39,43,46–48,51), fundamental aspects of this topic remain unclear. A particularly underexplored area of investigation is the identity of the phospho-amino acids in Upf1 critical for its ability to function in NMD. While studies on mammalian UPF1 have identified phosphorylated amino acids at the C-terminus (S-1078 and S-1096) (39), these residues are not present in *S. cerevisiae* Upf1, indicating they cannot have a universal role in NMD. In this report, we identified 11 phosphorylation sites in *S. cerevisiae* Upf1 (Figure 2A and B), including several that correspond to amino acids that are potentially phosphorylated in other species (Figure 3). Of particular note, the *S. cerevisiae* Upf1 phosphorylated residue, T-194, is also present in *H. sapiens* UPF1 and thus it will be of interest to determine whether this residue is also phosphorylated in human UPF1. Recently, Okada-Katsuhata and *et al.* (48) reported that human UPF1 T-28 is phosphorylated, a residue corresponding to S-6 in *S. cerevisiae* Upf1, a region not covered in our tandem MS/MS analysis (Figure 2B). It will be of interest to determine whether S-6 and any other amino acids besides the 11 we identified in *S. cerevisiae* Upf1 are phosphorylated under specific circumstances. Indeed, our finding that mutation of Y-742 to phenylalanine perturbed several Upf1 activities (Figures 6 and 7) raises the possibility that Y-742 is phosphorylated.

We demonstrated that a region comprised of 10 amino acids (amino acids 736–745) within the ATPase and helicase domain of Upf1 is essential for NMD activity (Figure 5C). While we did not identify all the critical amino acids required for the activity of this C-terminal motif, our mutation analysis revealed that the two tyrosine residues present in this motif—Y-738 and Y-742—have a role in Upf1’s NMD activity (Figure 6C). Mutation of these tyrosine residues to the phospho-mimetic, glutamic acid, caused the same degree of reduced NMD activity as mutation to phenylalanine, suggesting that phosphorylating these residues is not sufficient to allow them to function. This is consistent with the possibility that Upf1 must undergo a cycle of phosphorylation and dephosphorylation in *S. cerevisiae*, just as it does in higher eukaryotes (39,44,47,48,51,71). We suggest that Upf1 phosphorylation may trigger conformational changes that influence Upf1:Upf2 or Upf1:RNA interactions that are essential for NMD activity. We note, however, that we cannot rule out that the Tyr-to-Phe and Tyr-to-Glu alterations in the double-point mutants that we generated (Y738F/Y742F and Y738E/Y742E) decreased NMD activity by a mechanism independent of Upf1 phosphorylation. Our finding that neither mutation of Y-738 nor Y-742, alone, had measurable effects on Upf1 activities (Figure 6C, D and E) suggests that the phosphorylation at these two sites acts redundantly. Upf1 may be broadly buffered by such redundancy mechanisms to protect it from environmental and genetic insults.

Further investigation is required to identify the kinase(s) responsible for phosphorylating *S. cerevisiae* Upf1. In higher eukaryotes, Upf1 is phosphorylated at several serine/threonine-glutamine (S/TQ) motifs in the N- and C-terminal regions by SMG-1, a member of phosphatidylinositol 3-kinase-related protein kinases (39,48). Two recently identified NMD proteins, SMG-8 and SMG-9, promote this kinase activity (44,45,72) and two other NMD proteins, SMG-5 and SMG-7, promote the dephosphorylation of Upf1 (37,48,49,73–75). In *S. cerevisiae*, Ebs1, a protein similar in structure to human SMG-7 has been reported to be involved in NMD (76); however, no direct orthologs of SMG proteins in *S. cerevisiae* have been identified.

Upf1 has functions in addition to NMD, including promoting translational termination in both mammals and yeast (18–20,40,46,65,77,78). Upf1 serves to stimulate the accuracy of translation termination by suppressing translational read-through. In this article, we provide evidence that Upf1’s ability to promote translational termination fidelity depends on the same C-terminal phospho-motif important for its NMD activity (Figures 4 and 5). We also showed that the same tyrosine residues that promoted NMD activity—Y-738 and Y-742—are important for promoting translation termination efficiency (Figure 6E). Given the evidence that Upf1 promotes translation termination by virtue of its ability to interact with the translation release factors eRF1 (Sup45) and eRF3 (Sup35) (77,79), we propose that Upf1 phosphorylation might be necessary to dissociate release factors at the site of translation termination, as previously reported in mammals (36). By analogy, previous studies conducted in mammalian cells have shown that phosphorylated Upf1 interacts with the translation initiation factor eIF3 to suppress continued translation initiation (46).

The RNA-binding activity of Upf1 is known to be modulated by ATP (13,14,18,20). In the absence of ATP, Upf1 binds strongly to RNA, whereas ATP hydrolysis facilitates the dissociation of Upf1 from RNA (13,20). Recent studies have shown that the ATPase and helicase activities of Upf1 are stimulated upon binding of its CH-rich domain to Upf2, which, in turn, leads to decreased binding of Upf1 to the mRNA (21). Our finding that the Y738F, Y742F and Y738F/Y742F Upf1 mutants are deficient in ATPase activity (Figure 7B) raises the possibility that phosphorylation of Y-738 and Y-742 is important for such Upf1-driven ATP hydrolysis. Indeed, protein phosphorylation events are known to influence protein structural conformation, sub-cellular localization, molecular associations and enzymatic activities, including ATP hydrolysis (80–83). These results, coupled with the established literature, lead us to propose the following model for the role of Upf1 phosphorylation in NMD: PTC recognition by the translational machinery triggers Upf1 to bind to Upf2, leading to Upf1 phosphorylation, which, in turn, triggers its ATPase activity and subsequent Upf1:RNA disassembly and degradation of the released mRNA. While we regard this model as consistent with the available evidence, we stress that there is, as of yet, no direct evidence that Upf1 phosphorylation regulates Upf1 ATPase and helicase activities.

NMD has been considered as a potential therapeutic target for treating human genetic disorders caused by genes harboring nonsense or frameshift mutations generating PTCs (84–86). We suggest that a better understanding of the integral role of Upf1 phosphorylation in this mRNA surveillance pathway will aid in the development of novel approaches to treat such genetic disorders.
